# SR/RS Motifs as Critical Determinants of Coronavirus Life Cycle

**DOI:** 10.3389/fmolb.2020.00219

**Published:** 2020-08-21

**Authors:** Eleni Nikolakaki, Thomas Giannakouros

**Affiliations:** Laboratory of Biochemistry, Department of Chemistry, Aristotle University, Thessaloniki, Greece

**Keywords:** SR/RS motifs, SR protein kinases, coronaviruses, capsid assembly/disassembly, N protein

## Abstract

SR/RS domains are found in almost all eukaryotic genomes from *C. elegans* to human. These domains are thought to mediate interactions between proteins but also between proteins and RNA in complex networks associated with mRNA splicing, chromatin structure, transcription, cell cycle and cell structure. A precise and tight regulation of their function is achieved through phosphorylation of a number of serine residues within the SR/RS motifs by the Serine-Arginine protein kinases (SRPKs) that lead to delicate structural alterations. Given that coronavirus N proteins also contain SR/RS domains, we formulate the hypothesis that the viruses exploit the properties of these motifs to promote unpacking of viral RNA and virion assembly.

## Introduction

Coronaviruses (CoVs) have a single-stranded positive-sense RNA genome covered by an enveloped structure. The order of genes (5′–3′) contained in their RNA genome is as follows: replicase ORF1a/b, spike (S), envelope (E), membrane (M), and nucleocapsid (N) (McBride et al., 2014; Wu et al., 2020). The two-third of the RNA genome is covered by ORF1a/b encoding two overlapping replicase proteins in the form of polyproteins, which are then proteolytically processed by virally encoded proteases into the mature non-structural proteins, while the last third of the genone encodes the four structural proteins, S, M, E, and N and accessory proteins (Wu et al., 2020).

Of the four structural proteins, the nucleocapsid protein N is the one that is responsible for packaging the viral RNA (Chang et al., 2014; McBride et al., 2014). Many N proteins wrap and coil the RNA, thus keeping it stable inside the virus. Following infection, nucleocapsid uncoating takes place and N protein dissociates from viral RNA, while N protein association with RNA genome is a prerequisite for nucleocapsid assembly. Here we gather evidence from the literature and present a potential mechanism by which N protein-genomic RNA interactions may be governed by phosphorylation/dephosphorylation events spatially and temporally regulated.

## Structure of the N Protein

**FIGURE 1:**
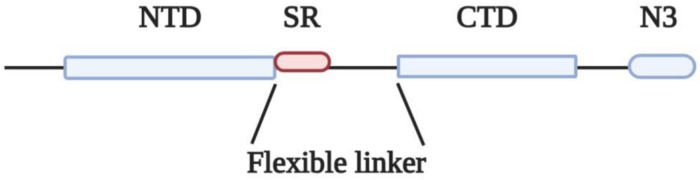
Domain organization of N protein. NTD, N-terminal RNA binding domain; SR, Serine/Arginine domain; CTD, C-terminal RNA binding domain; N3, C-terminal domain.

The N protein of coronaviruses is structurally organized into two domains, the N-terminal domain (NTD) and C-terminal domain (CTD), which are separated by a flexible linker (Figure 1). Both NTD and CTD have been shown to bind RNA through different mechanisms (Huang et al., 2004; Chen et al., 2007; Chang et al., 2009).

The flexible linkers of the different coronaviruses share very low sequence homology, yet they share some common properties: they are intrinsically disordered regions, they all contain a serine-arginine (SR)-rich domain (Table 1) and they are highly enriched in basic residues (Chang et al., 2009, 2014; Tylor et al., 2009). Based on these structural features, N proteins could be classified as SR/RS-domain containing proteins harboring also RNA Recognition Motifs (RRMs) (Birney et al., 1993; Nikolakaki et al., 2008). SR/RS regions are highly conserved sequences across species, while genes coding for SRPKs that are the main kinases phosphorylating serine residues within the SR/RS motifs, are found in all eukaryotes, even in single-celled organisms (Calarco et al., 2009; Giannakouros et al., 2011).

**TABLE 1:** SR/RS domains in N proteins of coronaviruses.

Coronavirus	SR/RS domain
SARS-CoV-2	SSRSSSRSRNSSR
SARS-CoV	SSRSSSRSRGNSR
MERS-COV	SSRASSLSRNSSRSSSQGSRS
Human coronavirus HKU1	SRPGSRSQSRGPNNRSLSRS
Human coronavirus NL63	RSNNSSRASSRSSTRNNSRDSSRSTSRQQSRTRS
Human coronavirus OC43	RSAPNSRSTSRTSSRASSAGSRSRAN
Human coronavirus 229E	SRAPSRSQSRSQSRGRGESKPQSR
Infectious bronchitis virus	RSGRSTAASSAAASRAPSREGSRGRRS
Transmissible gastroenteritis virus	SRDNSRSRSQSRSRSRNRSQSR
Murine hepatitis virus	RSAPASRSGSRSQSRGPNNRARSS
Bovine coronavirus	RSAPNSRSTSRASSRASSAGSRSR
Porcine hemagglutinating Encephalomyelitis virus	RSAPNSRSNSRAPNRTPSAGSRSR
HBV core protein*	RSPRRRTPSPRRRRSQSPRRRRSQSRESQC

*Hepatitis B virus is a partially double-stranded DNA virus but its core protein contains SR/RS dipeptides the impact of which on RNA binding and capsid assembly is discussed below in the text.

## Phosphorylation/Dephosphorylation of the SR/RS Domain Modulates Capsid Disassembly/Assembly

Viruses hijack cellular machineries in many ways to cause infection and to force the host cell to produce copies of the virus. In the context of this hijacking procedure the viruses take advantage of short linear motifs that have the capacity to encode a functional interaction interface and are found in intrinsically disordered regions of both cellular and viral proteins (Davey et al., 2011). These short motifs are aptly characterized by Davey et al. (2011) as an “Achilles’ heel” of the cell since they efficiently add functions to the limited viral genome. SR/RS dipeptides represent such a motif allowing the coronaviruses to utilize the cellular SRPKs to modulate the interaction of N protein with their RNA genome. As we have previously shown SR/RS domain-containing proteins share a common characteristic behavior in that they may occur in two different states: the aggregated state when they are unmodified and the soluble state when they are associated with RNA molecules or when the serine residues within the SR/RS domain are phosphorylated (Nikolakaki et al., 2008). Soluble proteins can switch from an RNA-associated to a phosphorylated form and vice-versa by a phosphorylation or respectively a dephosphorylation reaction, mediated by specific protein phosphatases, coupled with RNA association.

Following entry, the coronavirus genome needs to be translated by the cellular translational machinery to yield initially polyproteins pp1a and pp1ab and then through proteolytic processing the non-structural proteins that will drive viral genome replication and subgenomic mRNA synthesis (Sola et al., 2015; Fung and Liu, 2019). For translation to occur, nucleocapsid uncoating must take place. It has been previously shown for SARS-CoV that N protein is hypo-phosphorylated within the virion (Wu et al., 2009; Chang et al., 2014). Based on our previous data that phosphorylation by SRPK1 may dissociate the RNA molecules from an SR/RS-domain containing protein (Nikolakaki et al., 2008), and contrary to a previous suggestion that further dephosphorylation events would enhance uncoating of the viral RNA (Chang et al., 2013), we postulate that extensive phosphorylation by cellular SRPKs (SRPK1 and SRPK2) and potentially by additional cellular kinases may be the key event leading to displacement of N proteins from the viral RNA.

Coronavirus RNA-dependent RNA synthesis includes two distinct processes: genome replication, during which the coronavirus replicase synthesizes full-length negative “antigenomic” RNA which in turn serves as a template for the synthesis of new genomic RNA and transcription during which the replicase-transciptase complex synthesizes a set of negative sub-genomic RNAs which are then used as templates for the synthesis of positive-sense sub-genomic RNAs encoding the viral structural and accessory proteins (Sola et al., 2015). Both these processes are mediated by replication-transcription complexes which are assembled and anchored in Double-Membrane Vesicles (DMVs) derived from rearrangement of the cellular membranes mainly by the non-structural proteins nsp3, nsp4, and nsp6 (Fung and Liu, 2019). A critical issue is how the RNA genome is tethered to the newly translated replicase-transcriptase complex at the very early stages of infection. In this respect, Hurst et al. (2010) demonstrated that the SR region of N protein binds to nsp3. Of interest, this is an electrostatic interaction between the highly basic residues of the SR region and the highly acidic Ubl1-Ac segment of nsp3. According to the model proposed later by Hurst et al. (2013), N proteins must be displaced from the 5′ two-thirds of the incoming viral genomic RNA in order to allow translation, while residual nucleocapsid structure at the 3′ end of the genome would allow its immediate association with the newly synthesized nsp3. In this regard, we propose that phosphorylation by SRPKs at the very early stage of infection and the subsequent displacement of N protein molecules from the 5′ end of the viral RNA, in the form of an unzipping mechanism, would allow repeated rounds of genome translation, progressive formation of DMVs and as a result progressive assembly of replicase complexes in restricted loci of the endoplasmic reticulum. Yet, alongside the increase in nsp3 concentration, the SR regions of N proteins would dissociate from viral RNA and become attached to the acidic Ubl1-Ac segments of nsp3, while N proteins would remain associated with RNA through their NTDs and/or CTDs. This will lead to the progressive assembly of initiation complexes at the 3′ end of the viral genome, possibly with the participation of other viral non-structural and also cellular proteins, which would facilitate sub-genomic mRNA transcription (Zúñiga et al., 2010; Hurst et al., 2013). Phosphorylation may play a role in fine tuning the composition and function of these complexes. In fact it has been reported that phosphorylation of serine residues within the SR domain by glycogen synthase kinase-3 (and potentially by SRPK family members) is required for template switching. Phosphorylated SR domains recruit the RNA helicase DDX1 to the replication-transcription complex which facilitates template read-through and enables mainly genomic RNA synthesis (Wu et al., 2014).

**FIGURE 2:**
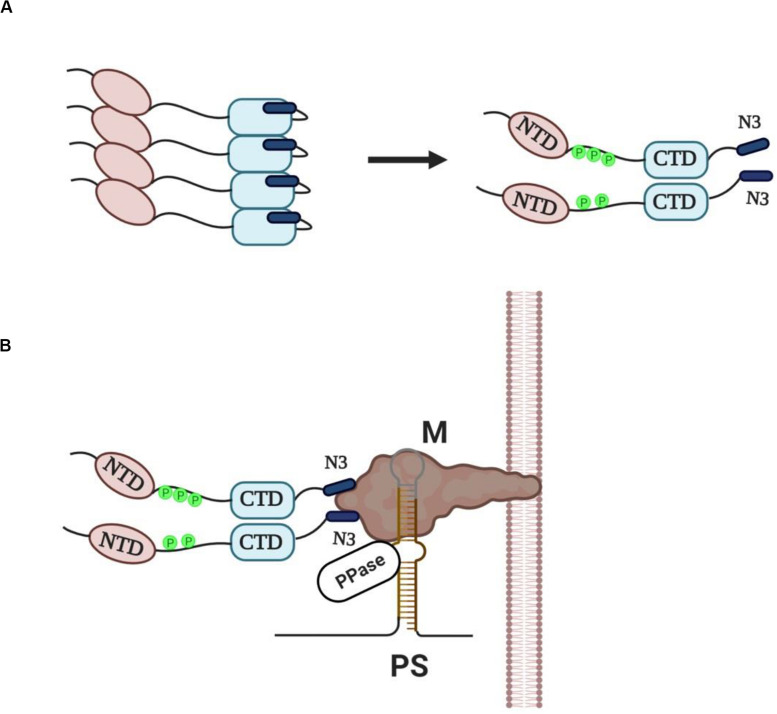
**(A)** Newly synthesized N proteins are prone to aggregation when they are unmodified (left panel). Phosphorylation of the SR/RS dipeptides by SRPKs (and possibly other cellular kinases) prevents aggregation and leads to the assembly of soluble dimers with the acidic carboxyl-tail domains (N3) exposed (right panel). **(B)** A provisional model of “encapsidosome” assembly. The exposed N3 domains interact with the M protein endodomain. The M protein endodomain binds also to the packaging signal (PS) of genomic RNA, while we propose that the genomic RNA-M protein complex carries a protein phosphatase activity (PPase). The phosphatase would dephosphorylate the SR/RS domains of the nearby associated dimers thus allowing the NTD and CTD interaction with genomic RNA. Furthermore, dephosphorylation would result in a conformational change of N proteins, dissociation of the N3 domains from the M protein endodomain and beginning of genomic RNA packaging. The dissociation of the N protein dimer from the M protein would leave space for a new N dimer to bind. The packaging process will go on with N protein dimers binding continuously to diverse sequences of genomic RNA until the entire RNA is enwrapped.

N protein is the most abundantly expressed protein during viral infection (Moreno et al., 2008) and is synthesized by cytosolic free ribosomes, while the other three structural proteins (S, M, E) and some membrane-associated accessory proteins are synthesized by ER-associated ribosomes as these proteins have to move along the secretory pathway (Masters, 2006). The ability of SARS-CoV N protein to self-interact was first demonstrated using yeast two-hybrid and co-immunoprecipitation experiments (He et al., 2004; Surjit et al., 2004; Luo et al., 2005). Mutational analysis revealed that the SR motif was responsible for this self-interaction, since deletion of this region largely abolished the N protein self-multimerization (He et al., 2004). Interestingly, SR/RS domain containing-proteins, and even those containing RRMs, are prone to aggregate under native conditions through their SR/RS domains (Yue et al., 2000; Nikolakaki et al., 2008), suggesting that the tendency to self-interact is not a unique property of coronaviruses N proteins but a common characteristic of SR/RS domain-containing proteins and could be attributed to their disordered structure. Insoluble aggregates not only would be non-functional in the virus assembly process but they could also jeopardize host cell viability. Even though Chang et al. (2013) proposed that phosphorylation may increase the oligomerization potential of N protein, it was previously shown that aggregation of SR/RS domain containing proteins can be prevented either by phosphorylation of the SR/RS domain or RNA association (Yue et al., 2000; Nikolakaki et al., 2008). Given the presence in the cytoplasm of infected cells of various viral sub-genomic as well as cellular RNAs, together with full-length viral genomic RNAs, and the fact that N protein is a non-specific nucleic acid-binding protein (Tang et al., 2005; Takeda et al., 2008) the scenario of RNA association is unlikely to happen because it would lead to encapsidation of random RNAs. On the other hand, Stohlman et al. (1983) were the first to show that MHV N protein acquires phosphates rapidly following its synthesis leading to a conformational change in the protein. Surjit et al. (2005) and Zakhartchouk et al. (2005) showed that the SARS-CoV N protein is phosphorylated at its SR region, while some years later Peng et al. (2008) demonstrated that SRPK1 was the responsible kinase and that phosphorylation reduced the self-association tendency of N protein. Furthermore, other reports indicated that N proteins were capable to form soluble dimers through their CTDs in the absence of RNA (Luo et al., 2005; Tang et al., 2005; Chen et al., 2007; Chang et al., 2013, 2014). We hence propose that N proteins are phosphorylated by SRPKs (and possibly other cellular kinases) and assembled into soluble dimers in which the proteins are kept together by the self-interacting ability of CTD but also by electrostatic interactions between stretches of negatively- (phosphorylated SR/RS domains) and positively-charged residues (non-phosphorylated SR/RS domains). Phosphorylated SR/RS domains not only prevent aggregation and allow escape from unfavorable interactions, such as with RNA molecules but also result in N protein unfolding and exposure of the acidic carboxyl-tail domain of the protein (N3) (Figure 2A).

Virus assembly occurs in the ER-Golgi intermediate compartment (ERGIC) and is orchestrated by the M protein (Masters, 2006, 2019). An electrostatic interaction, independent of viral RNA, between the N3 domain and a stretch of basic aminoacid residues located within the C-terminal domain of M protein (M endodomain) seems to be a critical step in the assembly process (Narayanan et al., 2000; Luo et al., 2006; Kuo et al., 2016; Masters, 2019). On the other hand, M protein was shown to specifically interact with a short packaging signal (PS) present only in the 5′ end of genomic RNA, thus selecting only genomic RNAs and excluding subgenomic mRNAs that lack the PS from the packaging process (Narayanan et al., 2003). The whole process may be envisaged as the assembly of an “encapsidosome,” analogously to the assembly of a spliceosome by phosphorylated SR splicing factors and mRNAs awaiting splicing (for a hypothetical model see Figure 2B). To note, while phosphorylation at multiple sites within the SR/RS domains by the SRPK (and the CLK) family of kinases is a prerequisite for spliceosome assembly, a dephosphorylation step is required for splicing to occur (Mermoud et al., 1994; Zhou and Fu, 2013). In this respect, SARS-CoV N protein was found dephosphorylated/hypophosphorylated within the virion (Wu et al., 2009, 2014), while Mohandas and Dales (1991) reported that a membranous-associated protein phosphatase exhibited high dephosphorylating activity against the N protein of neurotropic coronavirus JHM. Most effective would be a phosphatase activity associated with the PS containing genomic RNA-M protein complex, thus providing spatio-temporal control of the dephosphorylation procedure. In line with our hypothesis, M protein was recently found to interact with various phosphatase activities, such as protein phosphatase 6 catalytic subunit (PPP6C), protein phosphatase 1F (PPM1F), protein phosphatase 2A 65 kDa regulatory subunit A alpha isoform (PPP2R1A), protein phosphatase 2A 65 kDa regulatory subunit A beta isoform (PPP2R1B), protein phosphatase 2A 55 kDa regulatory subunit B alpha isoform (PPP2R2A), protein phosphatase 2B regulatory subunit 1 (PPP3R1) and protein phosphatase 6 regulatory subunit 3 (PPP6R3) (Gordon et al., 2020). A similar dephosphorylation event has recently been suggested for Hepatitis B virus core protein which also contains an SR/RS-rich domain and is a substrate of SRPK1 (Heger-Stevic et al., 2018). According to the proposed model, the phosphatase dephosphorylates only nearby Hepatitis B virus core protein dimers, and thus locally unleashes their RNA binding potential in proximity to packaging RNA. Similarly, a phosphatase activity associated with the M protein-genomic RNA complex would dephosphorylate the SR domains, and as a consequence allow the NTD and CTD interaction with genomic RNA. Such an interaction would also lead to a conformational change and release of the N3 domain from the M endodomain, leaving space for a new N dimer to bind. This would go on with further N protein dimers binding non-specifically to diverse sequences of genomic RNA until the encapsidation procedure is completed.

The nucleocapsid is then incorporated into a viral particle. Finally, coronavirus particles budded into the ERGIC are transported in smooth-wall vesicles and trafficked via the secretory pathway for release by exocytosis (Fung and Liu, 2019).

## Conclusion

Although the N proteins of different coronaviruses share low sequence homology, they all contain an SR/RS domain. A SARS-CoV genetic construct lacking the SR motif from the N gene showed defective replication and significantly reduced levels of infectious virions (Tylor et al., 2009), highlighting the importance of this region. We propose that SR/RS domain phosphorylation by SRPKs is an important step for nucleocapsid uncoating at the early stages of infection, while phosphorylation of the newly synthesized N proteins promotes their assembly in soluble dimers and transient oligomers and prevents association with non-specific RNAs. A dephosphorylation event spatially and temporally regulated is then required for virion assembly. Accordingly, a delicate fine-tuning of the phosphorylation/dephosphorylation procedure is essential to the viral life cycle and even small perturbations may have severe consequences. Considerable experimental work will be needed to substantiate this model. Of interest, a similar concept can also be adapted to other viruses since SR/RS motifs are found in several viral proteins (Nikolakaki et al., 1997; Lai et al., 2009; Heger-Stevic et al., 2018). In this respect, SRPIN340, an isonicotinamide compound that inhibits both SRPK1 and SRPK2 was shown to suppress virus propagation (Fukuhara et al., 2006), thus opening the possibility that SRPK inhibitors could be used as anticoronaviral therapeutics, especially since these inhibitors were recently shown to cause minimal or no harm to human cells (Tzelepis et al., 2018).

## Author Contributions

All authors listed have made a substantial, direct and intellectual contribution to the work, and approved it for publication.

## Conflict of Interest

The authors declare that the research was conducted in the absence of any commercial or financial relationships that could be construed as a potential conflict of interest.
